# Reply to “Letter to the Editor Re: Scott S.N., et al. Nutrients 2019, 11(5), 1022”

**DOI:** 10.3390/nu11112699

**Published:** 2019-11-07

**Authors:** Sam N. Scott, Lorraine Anderson, James P. Morton, Anton J. M. Wagenmakers, Michael C. Riddell

**Affiliations:** 1 School of Kinesiology and Health Science, York University, Toronto, ON M3J 1P3, Canada; mriddell@yorku.ca; 2 Independent Researcher, Toronto, ON M3J 1P3, Canada; lo.anderson16@gmail.com; 3 Research Institute for Sport and Exercise Sciences, Liverpool John Moores University, Liverpool L3 3AF, UK; j.p.morton@ljmu.ac.uk (J.P.M.); a.j.wagenmakers@ljmu.ac.uk (A.J.M.W.); 4 LMC Diabetes & Endocrinology, 1929 Bayview Avenue, Toronto, ON M4G 3E8, Canada

We appreciate the interest and comments from Dr. Jake Kushner regarding our recent review paper [1]. Here are our specific comments to his concerns.

Dr. Kushner highlights that our review incorrectly states that the American Diabetes Association (ADA) Standards of Medical Care in Diabetes endorses a specific recommendation for the distribution of calories among carbohydrates, fats, and proteins for people with diabetes. Dr. Kushner’s criticism is correct, and we thank him for pointing out that our suggestion in reference [1], that ADA endorses this recommendation is not right. The recent guidelines from the ADA [2,3] as well as the recently published consensus report on Nutrition Therapy for Adults with Diabetes or Prediabetes [4] have emphasized the importance of individualization of macronutrient distribution. The quotes Dr. Kushner provides from the 2018 Standards of Medical Care [2], stating that protein intake should be individualized according to the requirements of the individual, are also correct.

However, it is important to note that The National Academy of Medicine’s Dietary Reference Intakes still report the Adequate Macronutrient Distribution Range (AMDR) for carbohydrate as 45–65% of energy (http://nationalacademies.org/hmd/~/media/Files/Report%20Files/2019/DRI-Tables-2019/3_RDAAITWM.pdf?la=en). In our review paper [1], a better statement than the one in question would have been that “people with diabetes, on average, eat about the same proportions of macronutrients as the general public: approximately 45% of their calories come from carbohydrates, 36–40% of calories from fat and the remainder (15–19%) from protein [5,6,7]”.

We hope the above gives clarity to the issues raised by Dr. Kushner and we appreciate the opportunity to reply to the thoughtful comments made.

## References

[1] Scott S.N., Anderson L., Morton J.P., Wagenmakers A.J.M., Riddell M.C. (2019). Carbohydrate Restriction in Type 1 Diabetes: A Realistic Therapy for Improved Glycaemic Control and Athletic Performance?. Nutrients.

[2] Association A.D. (2018). 4. Lifestyle Management: Standards of Medical Care in Diabetes. Diabetes Care.

[3] Association A.D. (2019). 5. Lifestyle Management: Standards of Medical Care in Diabetes. Diabetes Care.

[4] Evert A.B., Dennison M., Gardner C.D., Garvey W.T., Lau K.H.K., MacLeod J., Mitri J., Pereira R.F., Rawlings K., Robinson S. (2019). Nutrition Therapy for Adults with Diabetes or Prediabetes: A Consensus Report. Diabetes Care.

[5] Delahanty L.M., Nathan D.M., Lachin J.M., Hu F.B., Cleary P.A., Ziegler G.K., Wylie-Rosett J., Wexler D.J. (2009). Association of diet with glycated hemoglobin during intensive treatment of type 1 diabetes in the Diabetes Control and Complications Trial. Am. J. Clin. Nutr..

[6] Vitolins M.Z., Anderson A.M., Delahanty L., Raynor H., Miller G.D., Mobley C., Reeves R., Yamamoto M., Champagne C., Wing R.R. (2009). Action for Health in Diabetes (Look AHEAD) trial: baseline evaluation of selected nutrients and food group intake. J. Am. Diet. Assoc..

[7] Oza-Frank R., Cheng Y.J., Narayan K.M., Gregg E.W. (2009). Trends in nutrient intake among adults with diabetes in the United States: 1988–2004. J. Am. Diet. Assoc..

